# Direct Observation Techniques Using Scanning Electron Microscope for Hydrothermally Synthesized Nanocrystals and Nanoclusters

**DOI:** 10.3390/nano11040908

**Published:** 2021-04-02

**Authors:** Natsuko Asano, Jinfeng Lu, Shunsuke Asahina, Seiichi Takami

**Affiliations:** 1 EP Business Unit, EP Application Department, SEM Team, JEOL Ltd., 3-1-2 Musashino, Akishima, Tokyo 196-8558, Japan; nasano@jeol.co.jp (N.A.); lujf@jeol.co.jp (J.L.); 2 Graduate School of Engineering, Nagoya University, Furo-cho, Chikusa-ku, Nagoya 464-8603, Japan; takami.seiichi@material.nagoya-u.ac.jp

**Keywords:** hydrothermal synthesis, scanning electron microscope, nanocluster, in situ observation, characterization

## Abstract

Metal oxide nanocrystals have garnered significant attention owing to their unique properties, including luminescence, ferroelectricity, and catalytic activity. Among the various synthetic methods, hydrothermal synthesis is a promising method for synthesizing metal oxide nanocrystals and nanoclusters. Because the shape and surface structure of the nanocrystals largely affect their properties, their analytical methods should be developed. Further, the arrangement of nanocrystals should be studied because the properties of nanoclusters largely depend on the arrangement of the primary nanocrystals. However, the analysis of nanocrystals and nanoclusters remains difficult because of their sizes. Conventionally, transmission electron microscopy (TEM) is widely used to study materials in nanoscale. However, TEM images are obtained as the projection of three-dimensional structures, and it is difficult to observe the surface structures and the arrangement of nanocrystals using TEM. On the other hand, scanning electron microscopy (SEM) relies on the signals from the surface of the samples. Therefore, SEM can visualize the surface structures of samples. Previously, the spatial resolution of SEM was not enough to observe nanoparticles and nanomaterials with sizes of between 10 and 50 nm. However, recent developments, including the low-landing electron-energy method, improved the spatial resolution of SEM, which allows us to observe fine details of the nanocluster surface directory. Additionally, improved detectors allow us to visualize the elemental mapping of materials even at low voltage with high solid angle. Further, the use of a liquid sample holder even enabled the observation of nanocrystals in water. In this paper, we discuss the development of SEM and related observation technologies through the observation of hydrothermally prepared nanocrystals and nanoclusters.

## 1. Introduction

In the last 30 years, many synthetic methods have been proposed and developed to produce nanomaterials with controlled sizes and morphologies [1,2,3]. The particle size, surface area, geometry, and chemical properties of the nanocrystals play a significant role in the interaction of these materials with biological systems, which affects their characteristics and applications in various disciplines [4,5,6]. Among the various synthetic methods, hydrothermal synthesis has gained attention and been subjected to research and development due to its capacity to produce small and shape-controlled nanocrystals in water [7,8]. Moreover, in situ surface modification during synthesis controls the surface of the nanocrystals and makes them easily dispersible in various solvents [8,9,10]. Generally, transmission electron microscopy (TEM) has been used to observe the size, shape, and heterogeneity of the nanocrystals. However, as the TEM images are obtained by projecting the signals on a screen, it is difficult to confirm the overlapping of nanoparticles. It is also difficult to observe the three-dimensional surface structure of nanocrystals from a two-dimensional projected image. On the other hand, scanning electron microscopy (SEM) uses the electrons that are emitted from the surface of samples. Therefore, SEM is a suitable method to visualize surface structures. One of the drawbacks of SEM is the spatial resolution of images. However, with the development of observation techniques at lower accelerating voltages and high voltage specimen bias, the resolution of SEM has been dramatically improved to directly observe the morphology and surface of nanocrystals, together with improvements in the lens detector [11,12,13,14,15,16]. In this study, we report the use of low-voltage high-resolution SEM and ultra-high solid angle energy-dispersive X-ray analyzer (EDS) to understand the 10–50 nm sized CeO_2_ nanocrystals and nanoclusters that were synthesized by a hydrothermal reaction.

## 2. Materials and Methods

### 2.1. Synthesis of CeO_2_ Nanoclusters

We have synthesized CeO_2_ nanoclusters using a flow-type reactor. The details are described in [17]. In short, the flow of preheated water was mixed with the flow of the precursor solution, that is, aqueous solutions of Ce(NO_3_)_3_ (0.01 M) with or without hexanedioic acid (0.05 M) at a junction in the tubular reactor. The reaction temperature was set to 250 °C, and the pressure was maintained at 25 MPa. The typical reaction time was 1.9 s. The product solution was depressurized with a backpressure regulator and collected at the outlet. The products were washed and dried.

### 2.2. Synthesis of Organic Ligand-Modified CeO_2_ Nanocrystals

Surface-modified CeO_2_ nanocrystals were synthesized in supercritical water, according to previous reports [18]. Briefly, cerium hydroxides (2.5 mL of 0.05 M cerium hydroxides) and 0.09 g of decanoic acid were transferred to a pressure-resistant reactor, and the reaction was performed at 400 °C for 10 min. The obtained organic ligand-modified CeO_2_ nanoparticles were washed with ethanol and toluene. The products were dispersed in 10 mL cyclohexane and were freeze-dried.

Silicon (100) substrates were modified with organic molecules to assemble the modified CeO_2_ nanocrystals on them. First, the silicon substrates were treated with ozone for 30 min to produce hydroxylated silicon oxide. Next, the silicon substrate was immersed in a solution of 1.15 g 3-aminopropyltriethoxysilane (3APTS), 1.26 mL 28% ammonia aqua, 45.6 mL ethanol, and 0.76 mL water to produce amine group termination. To further attach APTS to the substrate, the substrates were heated at 130 °C for 2 h. Then, the substrate was immersed in 5 mL N,N-dimethylformamide (DMF) containing 0.2 M of 3,4-dihydroxyhydrocinnamic acid (DHCA), 0.2 M of 1-ethy1-3-(3-dimethylaminopropyl) carbodiimide hydrochloride (EDC), and 0.02 M of N,N-dimethy1-4-aminopyridine (DMAP) for 15 h to produce catechol termination on the surface of substrates. Finally, the surface-modified silicon substrates were immersed in 0.2 mL cyclohexane containing 1 mg of organic ligand-modified CeO_2_ nanocrystals and then sonicated for 1 h to immobilize the CeO_2_ nanocrystals on the substrates. Subsequently, the substrates were rinsed with fresh cyclohexane solvent and dried.

### 2.3. Synthesis of Composite of Mesoporous SBA-15 and CeO_2_

SBA-15 and mesoporous CeO_2_ were synthesized using the following procedure [19]. First, 5.14 g of Pluronic P123 was dissolved in a mixture of 30.96 g of aqueous 37% HCl and water (144 g). Then, 11.12 g of tetraethoxysilane (TEOS) was added to this solution and stirred for 20 h at 40 °C. The mixture was then transferred to a stainless steel reactor with a Teflon lining and heated for 24 h at 100 °C. After cooling, the mixture was filtered, washed, and air-dried. Finally, mesoporous SBA-15 was obtained by calcining the mixture at 550 °C for 5 h. 

Next, the obtained SBA-15 was used as a solid template to prepare mesoporous CeO_2_. SBA-15 (0.50 g) and Ce(NH_4_)_2_(NO_3_)_6_ (0.5 g) were dispersed in ethanol (5.0 mL). The mixture was stirred at 50 °C until the ethanol was completely removed. The dried mixture was calcined in air at 450 °C for 5 h.

### 2.4. Low-Voltage SEM

**Figure 1:**
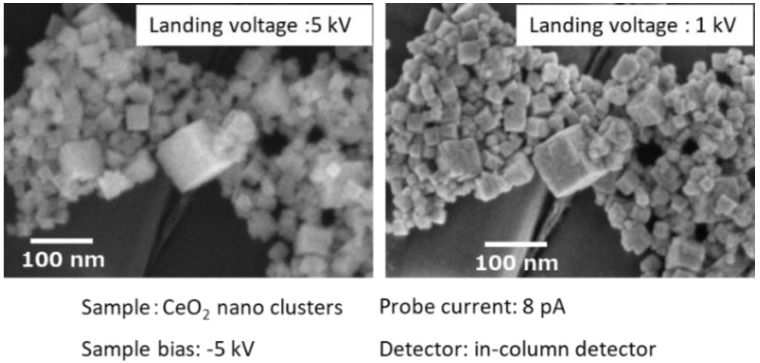
SEM images of CeO_2_ nanoclusters with different electron landing voltages.

The development of low-voltage (LV) field emission (FE) SEM realized a spatial resolution of 1 nm or less at an acceleration voltage of 1 kV. Furthermore, by choosing appropriate observation conditions, different information, such as material topology and composition, are also selectively obtained. In this report, we focused on the observation of extremely low impact electron energy to collect information from the surface. The mean free path of an electron with an energy of 100 eV in a solid sample is below 1 nm [14]. Therefore, the observation of the surface morphology at low electron energies is favorable. However, this low electron energy resulted in a larger diameter of the probe due to the chromatic aberration (Cc). The diameter of the electron probe size (dc) is described as dc = Cc(ΔV/V_acc_)α, where ΔV is the energy spread, V_acc_ is the acceleration voltage, and α is the angle of the beam(rad) [20]. Therefore, smaller ΔV and Cc values are necessary to minimize the probe diameter. Currently, we can use combined electrostatic and magnetic lenses to minimize Cc as well as field emission-type emitters with small ΔV. These so-called super hybrid lenses (SHL) are equipped on a FE-SEM (JSM-IT800SHL, JEOL, Tokyo, Japan). In addition, a negative surface potential can be applied to the sample surface to lower the impact electron energy, which reduces the scattering of electrons in the sample. Therefore, the lowest impact electron energy to the surface is reduced to 10 eV, while maintaining a small probe size with high coherency. This low landing voltage technique also suppressed the charging up and damage of the sample, leading to a clear observation of the shape and size of the nanocrystals and nanoclusters. Figure 1 shows an SEM image of CeO_2_ nanoclusters observed at a landing voltage of 1 kV (sample bias: −5 kV, probe current: 8 pA, detector: in-column detector). The shape and size of the CeO_2_ nanocrystals were clearly observed at both landing voltages. However, the image at 1 kV landing voltage had much finer details of the surface steps because of the smaller penetration depth.

### 2.5. Cross Section Polisher

**Figure 2:**
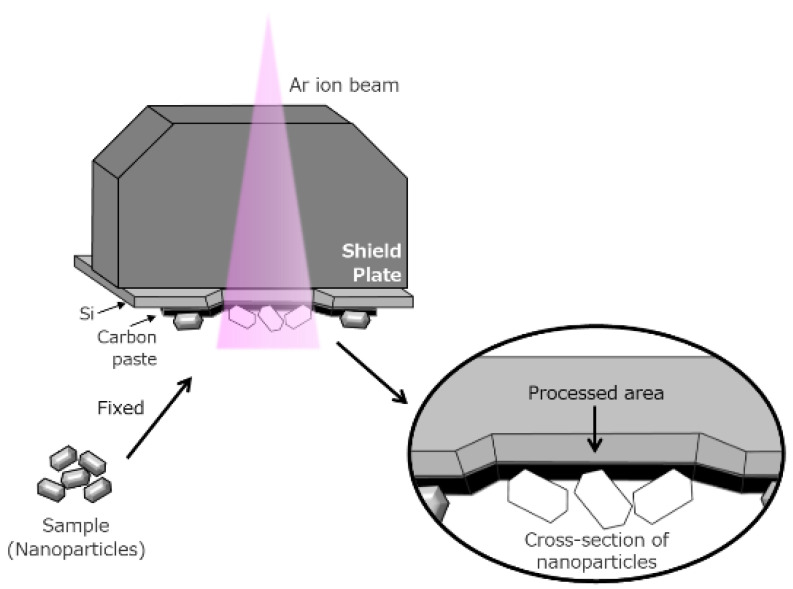
Precise cross sectioning using broad Ar ion beam.

A novel sample preparation technique for SEM observation of these materials was also developed. We used a cross section polisher (IB-19520CCP, JEOL, Tokyo, Japan) to irradiate the sample with an Ar ion beam to display the internal structures without causing serious artificial effects. The design of this technique is shown in Figure 2. A shield plate was placed on top of the specimen to determine its position. Only the portions left uncovered by the shield plate were milled using an Ar ion beam.

### 2.6. Multi-Energy Dispersive X-ray Spectroscopy (EDS)

The recent development of a silicon drifted detector (SDD) detector significantly enlarged the detection area of characteristic X-rays, which enabled a large solid angle for X-ray detection. The SDD also improved the electronic noise and dynamic range. Owing to these improvements, more characteristic X-rays can be detected, which allowed the detection of faint characteristic X-ray signals with improved signal to noise (S/N) ratio during SEM measurement. Thus, the necessary acquisition time for EDS mapping is shortened dramatically. In this experiment, we employed a multi-EDS system with a total detection area of 440 mm^2^, with a total solid angle of more than 0.15 sr from Oxford Instruments.

### 2.7. In Situ Holder

**Figure 3:**
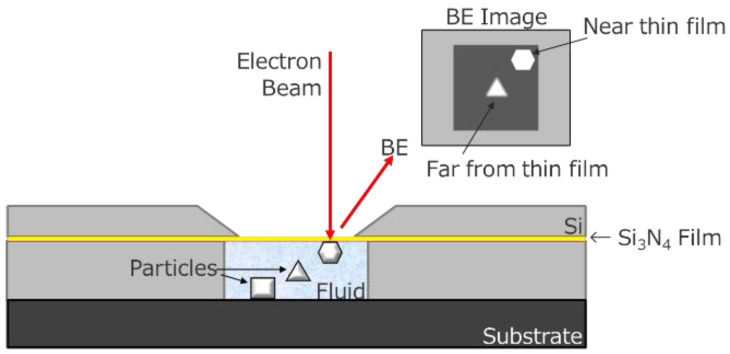
Schematic draw of in situ holder.

In most cases, SEM observation is performed in vacuo because electrons are scattered by molecules in air or in water. Therefore, the observation of nanomaterials in water remains difficult. Recently, we attempted to use an in situ holder (FlowVIEW Tek) with a 30 nm thick Si_3_N_4_ window. Figure 3 shows a schematic of the in situ holder. This holder enables SEM observation of nanomaterials in water by detecting electrons penetrated through the window [21]. This window has sufficient strength to hold water during the SEM observation. Si_3_N_4_ is composed of lighter elements; thus, electrons are less scattered by this window.

## 3. Results and Discussion

**Figure 4:**
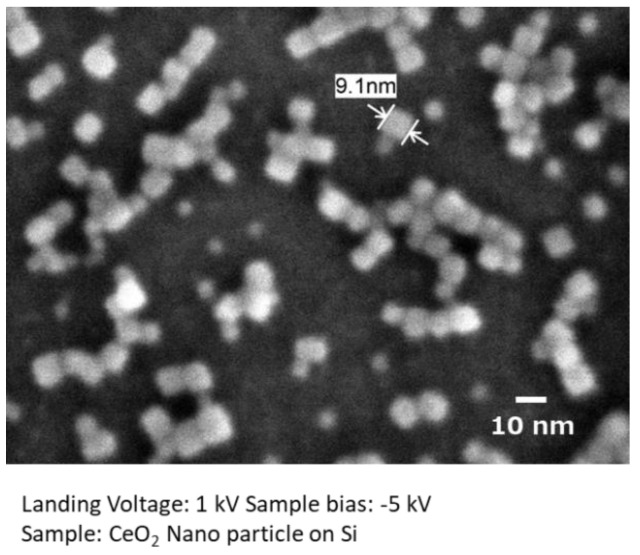
Low-voltage high spatial resolution imaging.

Figure 4 shows a high spatial resolution image of hydrothermally produced CeO_2_ nanocrystals on a Si substrate. The cubic shape of the surface-modified CeO_2_ nanocrystals was well resolved, even at several nanometers. This image was taken with a landing energy of 1 keV, and the sample bias was set to −5 kV. It is clearly confirmed that the low-landing voltage technique visualizes more details of the samples.

**Figure 5:**
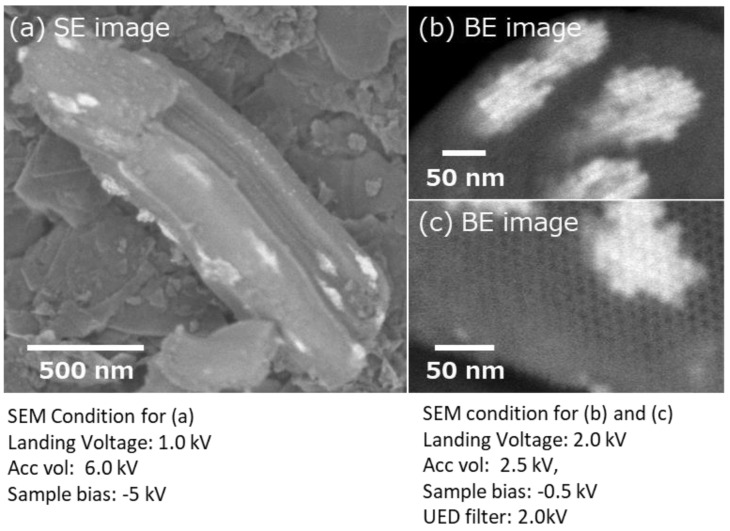
SEM images of mesoporous silica SBA-15 embedded with nano-casted CeO_2_. (**a**) Surface image of mesoporous silica SBA-15 which has been nano-casted with CeO_2_. (**b**) Cross section of longitudinal direction for CeO_2_ in meso pores. (**c**) Cross section of CeO_2_ in meso pores.

This technique is also applicable to mesoporous materials. Figure 5a shows SBA-15 mesoporous silica, where CeO_2_ was nano-cast. The SEM image was obtained with a landing voltage of 1 kV and sample bias of −5 kV. Because of the reduced landing voltage, CeO_2_ nanoclusters on or just below the surface of SBA-15 were selectively visualized as lighter contracts [16]. Moreover, to observe the CeO_2_ nanoclusters located deep inside SBA-15, the sample was processed using an Ar ion beam cross-sectional polisher (Figure 2). Cross-sectional images are shown in Figure 5b,c. During the observation of the cross section, an energy filter (2 kV) was used to selectively collect the back-scattered electron signals. The landing voltage was set to 2.0 kV, and a −0.5 kV bias voltage was added to the sample. The SEM images showed that the hexagonal mesochannels of SBA-15 were clearly confirmed from the cross section. At the same time, many CeO_2_ nanoclusters were confirmed in the mesopores of SBA-15. The shape and size of the prepared CeO_2_ nanocrystals were in good agreement with those of the pores of mesoporous silica used as the template. From the longitudinal directional cross section image (Figure 5b), the distribution of CeO_2_ clusters in SBA-15 and their sizes can be estimated. 

**Figure 6:**
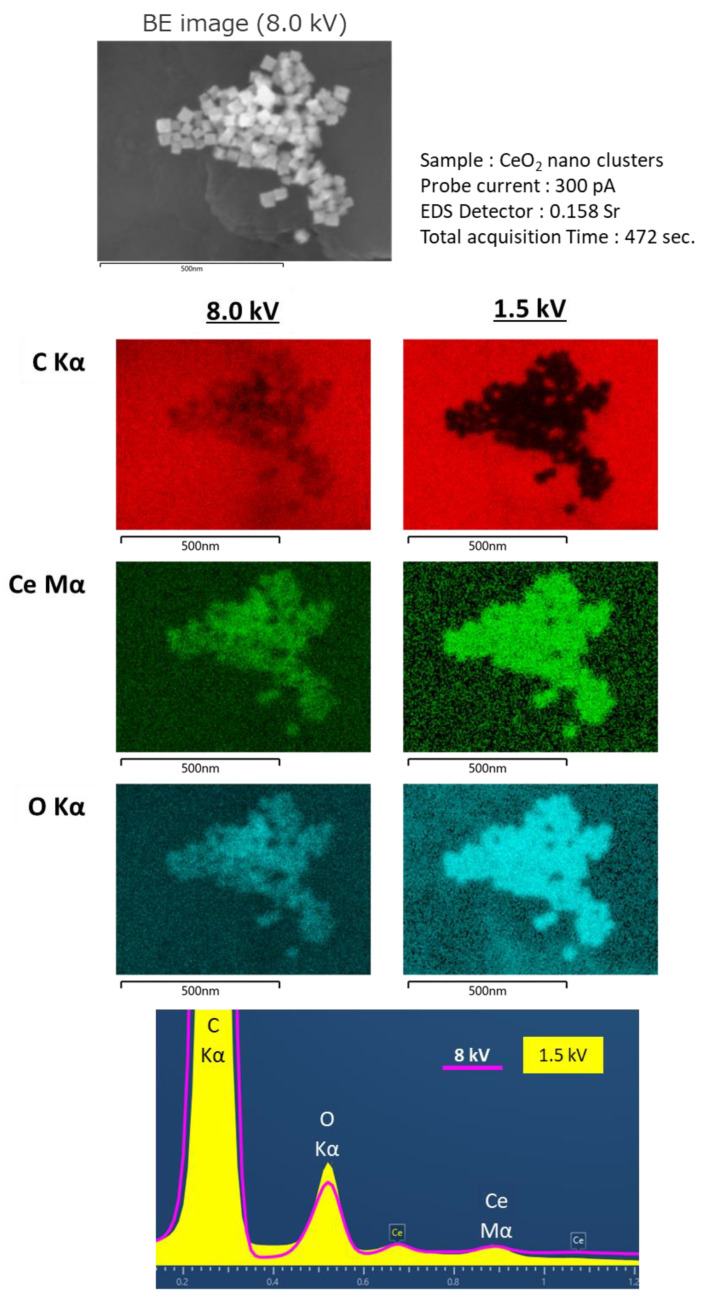
Results of large solid angle Energy Dispersive X-ray Spectroscopy (EDS).

Furthermore, the effect of the low-voltage technique was remarkable in the EDS analyses. Generally, high electron probe currents are required to generate characteristic X-rays from samples for EDS analyses. SEM images of the CeO_2_ nanocrystals (Figure 6) were obtained with a landing voltage of 8 kV. The elemental maps of Ce and O were clearly visible. However, the elemental map of C was blurred because the incident electrons were not fully scattered by the CeO_2_ nanocrystals with sizes less than 50 nm. The penetrated electrons interacted with the carbon substrate under CeO_2_ and emitted characteristic X-rays. As a result, X-ray signals were detected from the entire area of the carbon substrate and formed an EDS map of C with a slight view of the nanocrystals. On the other hand, the electrons with a lower landing voltage (1.5 kV) could only interact with the surface of the sample to emit the characteristic X-rays. Therefore, the EDS map of C did not show any signals from the substrate under the nanocrystals. 

**Figure 7:**
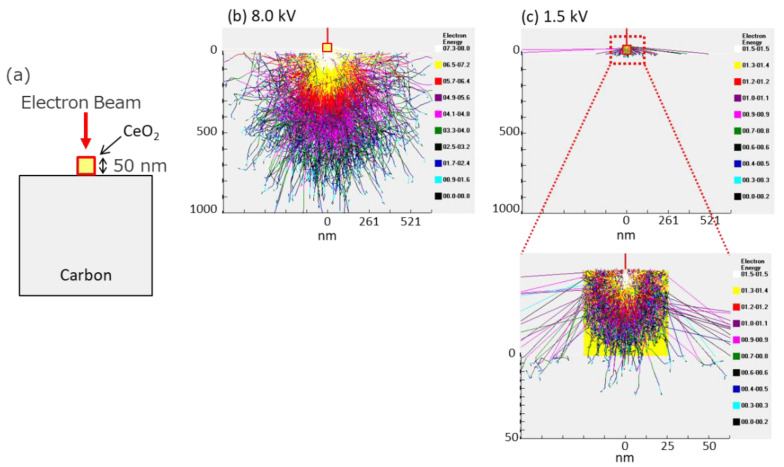
Calculation of electron penetration depth in 50 nm CeO_2_. (**a**) A schematic image of this simulation. (**b**,**c**) Results of simulations in different acceleration voltages, 8.0 kV, 1.5 kV.

The simulation results of the electron penetration depth in 50 nm CeO_2_ nanocrystals are also shown in Figure 7. The penetration depth of electrons with a landing voltage of 1.5 kV is less than 50 nm. This indicates that even for the EDS analysis, the low-voltage technique can also promote the selective acquisition of surface information. 

**Figure 8:**
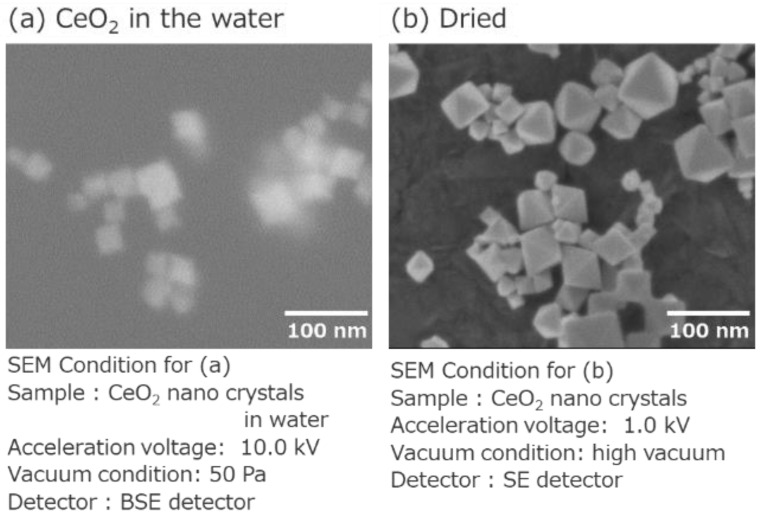
BSE image of in situ observation for CeO_2_ in water. (**a**) An image of the CeO_2_ nanocrystals in water. (**b**) An image of the dried CeO_2_ nanocrystals.

Figure 8a shows an image of the CeO_2_ nanocrystals in water. Usually, nanomaterials which are smaller than 50 nm in water cannot be observed using SEM. However, as shown in Figure 3, the use of an in situ holder enabled observation. As confirmed in Figure 8a, the size and dispersive of the CeO_2_ nanocrystals in water were visualized. By comparing the SEM images obtained in vacuum (Figure 8b), we noticed that the measurement of nanomaterials in water well reproduced the size and dispersive of the CeO_2_ nanocrystals.

## 4. Conclusions

Various shapes of hydrothermally synthesized CeO_2_ nanocrystals and nanoclusters were successfully observed using recent SEM techniques. SEM images are sensitive to the surface structure of the samples, and thus the detailed morphology that related to crystal growth at the nanometer scale can be visualized. Furthermore, the cross-sectional images of mesoporous silica embedded with CeO_2_ nanoparticles clearly show how the nanoparticles were produced in the mesopores. In addition, by using an in situ holder with an ultrathin Si_3_N_4_ window, CeO_2_ nanocrystals in water were directly observed. It is possible to directly observe the structure that is thought to exhibit the function of the nanoclusters of CeO_2_ synthesized under hydrothermal conditions by using recent SEM techniques. Most researchers are currently using TEM for the analysis of nanocrystals and nanoclusters. However, the development of SEM with improved performance and usability enabled us to obtain the high-resolution images of hydrothermally synthesized nanoparticles. In addition to the size and morphology, the surface structure can be clearly confirmed for the samples with the size of 10–100 nm. Moreover, it is possible to perform high-quality elemental analysis when the particle size is larger than 50 nm. By providing high-resolution imaging and high-quality analysis, SEM can now deepen the understanding of the essence of nanotechnology.

## 5. Patents

The cross section method which was used patented as JP 4922632.

## Data Availability

Not applicable.
